# Evidence for contact calls in fish: conspecific vocalisations and ambient soundscape influence group cohesion in a nocturnal species

**DOI:** 10.1038/srep19098

**Published:** 2016-01-11

**Authors:** L. van Oosterom, J. C. Montgomery, A. G. Jeffs, C. A. Radford

**Affiliations:** 1 Leigh Marine Laboratory, Institute of Marine Science, PO Box 349, Warkworth, 0941, New Zealand

## Abstract

Soundscapes provide a new tool for the study of fish communities. Bigeyes (*Pempheris adspersa*) are nocturnal planktivorous reef fish, feed in loose shoals and are soniferous. These vocalisations have been suggested to be contact calls to maintain group cohesion, however direct evidence for this is absent, despite the fact that contact calls are well documented for many other vertebrates, including marine mammals. For fish, direct evidence for group cohesion signals is restricted to the use of visual and hydrodynamic cues. In support of adding vocalisation as a contributing cue, our laboratory experiments show that bigeyes significantly increased group cohesion when exposed to recordings of ambient reef sound at higher sound levels while also decreasing vocalisations. These patterns of behaviour are consistent with acoustic masking. When exposed to playback of conspecific vocalisations, the group cohesion and vocalisation rates of bigeyes both significantly increased. These results provide the first direct experimental support for the hypotheses that vocalisations are used as contact calls to maintain group cohesion in fishes, making fish the evolutionarily oldest vertebrate group in which this phenomenon has been observed, and adding a new dimension to the interpretation of nocturnal reef soundscapes.

There are many examples of vertebrates that use vocalisations as contact calls between spatially separated members of a group in order to maintain group cohesion. This occurs particularly in animal groups where individuals or subgroups move beyond the visual range of other group members^1^. The acoustic complexity and encoded information of contact calls can vary between, and within animal groups, as can the target (i.e.; mates, kin or group members) and the purpose (i.e.; caller identity, confirming location or distance)^2^. Chimpanzees, *Pan troglodytes*, produce a pant-hoot contact call targeted at maintaining contact with allies and associates^3^. Contact calls are also used to reduce the chance of separation from the group, such as in juvenile black caiman, *Melanosuchus niger*, which vocalise on or near water banks to increase group cohesion between siblings^4^. African elephants, *Loxodonta africana,* are able to produce infrasonic sound that can be detected by conspecifics up to 2.5 kilometres away^5^, which can convey important information about caller identity for familial relations^6^, or be used to coordinate directional movement of visually isolated subgroups, or enable the maintenance of loose group cohesion^5^,^7^. Contact calls may vary with proximity of group members, such as in Hawaiian spinner dolphins, *Stenella longirostris,* which produce contact whistles when in a dispersed group, but will switch to burst pulses for communication between animals in closer proximity^8^.

Bony fish (teleosts) are an extremely diverse vertebrate clade, and correspondingly have evolved the largest diversity of sonic organs among vertebrates^9^. They produce sound by either mechanically vibrating or stridulating rigid or bony appendages or using specialised sonic muscles coupled with a swim bladder^10^,^11^. The variety of mechanisms produces vocalisations that vary temporally and spectrally and are characteristically species-specific^12^,^13^,^14^. Although fish are known to vocalise during agonistic interactions^15^, and during courtship and other reproductive activities^16^, currently only anecdotal evidence exists for the possible use of contact calls^17^,^18^,^19^.

Social grouping to avoid predation and increase foraging success^20^ is widespread across vertebrate groups. Many fishes form social groups, or shoals, and some exhibit highly coordinated schooling behaviour^20^. Known stimuli mediating normal group coordination include visual and hydrodynamic cues^21^. Hydrodynamic cues are intrinsically short range, and visual cues become limited in low light, so it has been suggested that vocal cues would increase in importance in nocturnal species^17^,^18^. The consideration of soundscape partitioning, or a reduced overlap in the frequencies of vocalisations emitted by species, also provides independent evidence for an increased importance of nocturnal vocalisations in fish^22^.

The New Zealand bigeye, *Pempheris adspersa,* are abundant along New Zealand’s north-east coast and exhibit interesting diurnal movement and vocal behaviour. During the day they take refuge in caves and outcrops and by night they leave their caves to forage along the nearby reef in loosely-knit shoals, returning to their shelters around dawn^23^. Bigeyes actively vocalise both day and night, emitting a ‘pop’ call with a mean peak frequency of 405 ± 12 Hz and mean duration of 7.9 ± 0.3 ms^18^. While it is thought that all fishes can detect low frequency sounds through inner ear function (otoliths)^10^, some groups have evolved ancillary hearing structures that increase their ability to detect sound^10^. The Baudelot’s ligaments found in bigeyes connect the swim bladder to the inner ear and lateral line (otolaterophysic connection), increasing hearing sensitivity to the frequency bandwidth of conspecific vocalisations by up to 20 dB^24^. Recent work by Radford, *et al.*^18^ estimated the minimum and maximum active space of bigeye vocalisations to be between 0.6 m to 31.6 m under realistic ambient sound variations, such as those that occur with time of day, moon phase, and season. The active vocal behaviour and auditory specialisation indicate a biological importance of vocalisations in this species, and the large active space of the call along with their nocturnal shoaling behaviour suggest their vocalisations could be of use as a contact call.

Therefore, the aim of this study was to experimentally test whether vocalisation plays a role in group cohesion of fishes, using the New Zealand bigeye as the test species. The experiment involved assessing changes in conspecific spacing and vocal behaviour in groups of captive bigeyes when exposed to varying levels of ambient sound, or the playback of conspecific vocalisations. When avoidance of elevated ambient noise levels is impossible, animals, including fish, may compensate by altering their behaviour^25^,^26^ or the acoustic properties of their signal to compensate for the loss of auditory sensitivity^27^,^28^. If bigeye vocalisations are used as contact calls, it is hypothesised that an increase in ambient noise would lead to an adjustment in proximity of individuals within shoals to improve auditory reception, or a change in vocal behaviour, such as increased level or changed frequency of vocalisation. Almost all research into contact calls in vertebrates has shown an attraction of individuals to conspecific vocalisations^2^, thus it was hypothesised that playback of conspecific vocalisation to bigeyes would also elicit changes in group cohesion or vocal behaviour.

## Results

### Ambient sound playback

**Figure 1:**
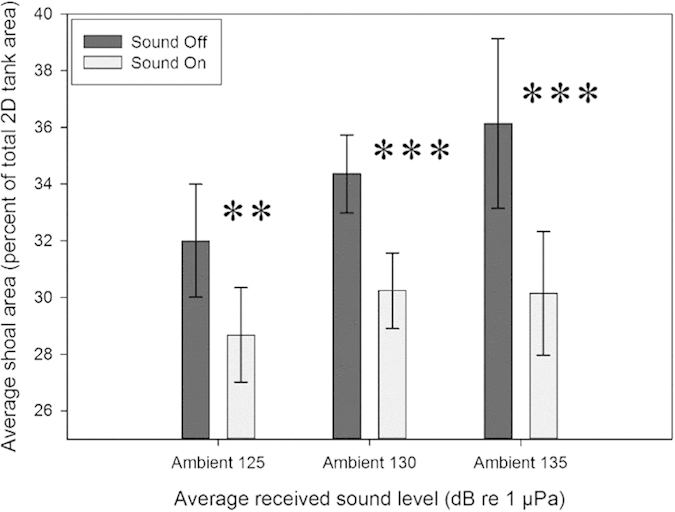
Mean percent of total tank area occupied by bigeye shoal during silent control (sound off) periods and during exposure to ambient sound at three average received levels (125, 130 and 135 dB re1 μPa). ** indicates statistical significance *p* < 0.01 *** indicates statistical significance *p* < 0.001.

Shoal area decreased significantly when ambient sound was played at 125 dB re 1 μPa resulting in a 3.3% decrease (t_27, 0.05_ = 4.0; P < 0.01), 4.1% decrease at 130 dB re 1 μPa (Z = −4.3; P < 0.001) and 6.0% decrease at 135 dB re 1 μPa (Z = −3.9; P < 0.001) compared to sound-off (Fig. 1).

**Figure 2:**
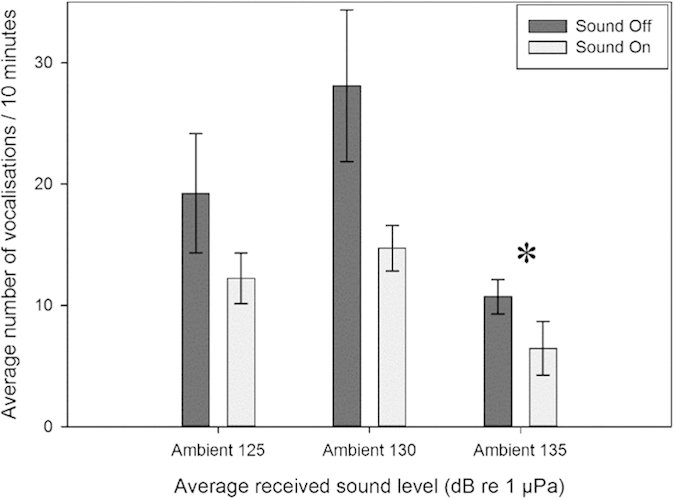
Mean number of bigeye vocalisations recorded per shoal during silent control (sound off) periods and during exposure to ambient sound of three average received levels (125, 130 and 135 dB re 1 μPa). * indicates statistical significance *p* < 0.05.

Bigeye vocal behaviour, measured as number of vocalisations in a 10 minute period (vocalisation 10 min^–1^), changed with increasing ambient playback levels during sound-on periods compared to corresponding sound-off periods, with the largest decrease in vocalisation rates tending to occur at 130 dB (Fig. 2). However, there was only a significant difference for ambient sound played at an average received level of 135 dB re 1 μPa (Z = −2.4; P = 0.016) with a decrease of only 4.3 calls per shoal per 10 minutes.

### Bigeye vocalisation playback

**Figure 3:**
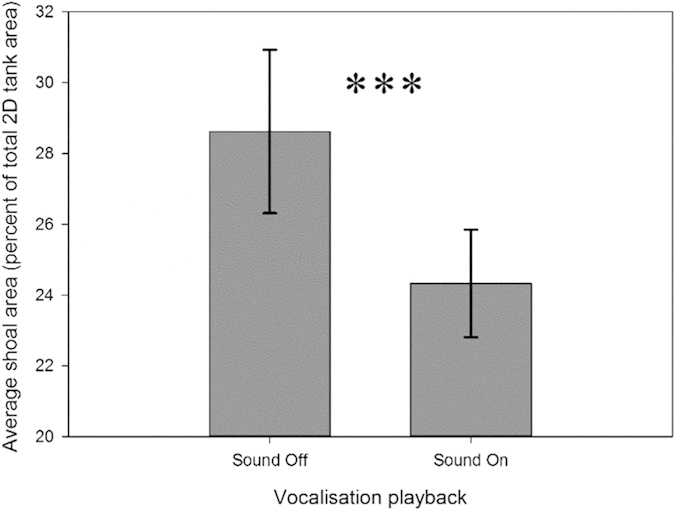
Mean percent of total tank area occupied by bigeye shoal during silent control periods and during exposure to vocalisation playback (average received level of 135 dB re 1 μPa). *** indicates statistical significance *p* < 0.001.

When conspecific vocalisations were played to fish, shoal area decreased by 4.3% (Z= −3.894; P < 0.001) compared to sound-off (Fig. 3).

**Figure 4:**
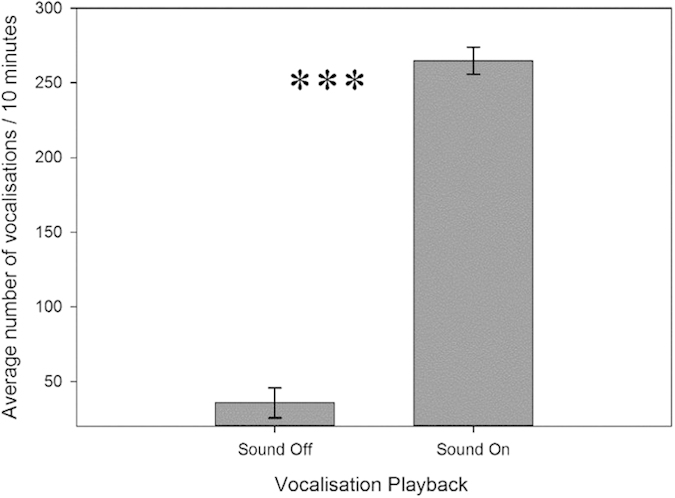
Mean number of bigeye vocalisations recorded per shoal during silent control periods and during exposure to vocalisation playback (average received level of 135 dB re 1 μPa). *** indicates statistical significance *p* < 0.001.

Playback of bigeye vocalisations had the opposite and a much larger effect on bigeye vocal behaviour than ambient sound playback, with vocalisations increasing in the presence of playback of bigeye vocalisations by an average of 229 calls per shoal per 10 minutes (Z = −4.6; P < 0.001) (Fig. 4).

## Discussion

**Figure 5:**
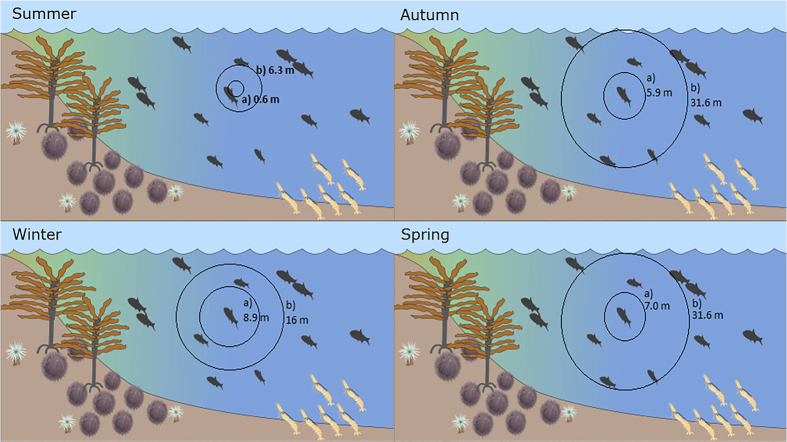
Representation of the effective calling range of bigeye vocalisation during. **(a)** new moon and (**b**) full moon based on data from Radford *et al.*^18^.

This study provides the first experimental evidence in support of contact calls being used for maintaining group cohesion in teleost fish. Captive shoals of bigeye significantly increased group cohesion when exposed to recordings of ambient reef sound at higher sound levels while also decreasing vocalisations. These patterns of behaviour are consistent with maintaining group cohesion via vocalisation in the presence of increased auditory masking. The present study showed that as ambient sound levels increased the distance between individual fish decreased, which would be an effective behavioural response to overcome the decreased active space caused by masking from elevated ambient sound^18^ (Fig. 5). When exposed to playback of conspecific vocalisations, the group cohesion and vocalisation rates of bigeyes both significantly increased.

Ambient sound from natural or anthropogenic sources varies spatially and temporally and when avoidance of elevated noise levels is impossible animals using acoustic signals are known to compensate behaviourally, often by adjusting spatial distribution or increasing signal amplitude^26^,^27^,^28^,^29^. Some birds, such as the silvereye, *Zosterops lateralis*, can change the frequency of emitted signals to increase detection above background noise^30^. Other animals increase the amplitude of their calls, such as the North Atlantic right whale, *Eubalaena glacialis*^28^. This increase in signal amplitude, the Lombard Effect, was recently found in fishes for the first time in the blacktail shiner, C*yprinella venusta*, which increases the amplitude of their vocalisations when exposed to high levels of ambient sound^27^. This was difficult to test in the current experiment as variation in amplitude could result from differences in the proximity of callers to the hydrophone, or could be an artefact of the ambient sound played in the tank overlapping with the upper frequencies of bigeye vocalisations. However, the number of vocalisations was measurable and bigeyes were found to decrease the number of calls emitted in response to elevated ambient sound. This decrease was only significant for the highest of the three ambient levels (135 dB re 1 μPa), due to high variation in the number of calls produced by each shoal during sound treatment and silent controls. Variation in calling rates is common under experimental and natural conditions; ambient sound playback differentially affects the calling rate of amphibians^31^ and the rate of contact calls produced by some primates depends on group spread and travelling speed^32^. It is possible that the consistent increase in group cohesion of bigeyes decreased the need for some fish to call as frequently, as they may have moved within range of other methods of maintaining shoal cohesion, such as hydrodynamic detection.

Playback of conspecific vocalisations to captive shoals of bigeyes also produced a pronounced decrease in shoal area compared to silent controls. An attraction of individuals to the contact calls of conspecifics is common among vertebrates^2^. When individuals become isolated, Guinea baboons^33^ and spider monkeys^34^ produce contact calls to promote reunions. Bats attract or repel group members using contact calls that are spectrally less complex than their harmonic echolocation calls^35^,^36^. Capybaras, produce non-harmonic ‘clicks’ that promote alertness and elicited approach to the direction of the call^37^. Unlike more complex calls of larger animals that contain information about individual identity, such as elephants^6^, these simple bigeye vocalisations do not appear to vary among individuals and may only code for species recognition. While the information coded in the contact call of bigeyes is unknown, the remarkably consistent production of sound during nocturnal activity, and the positive phonotaxy demonstrated here suggest it is highly likely they are used as contact calls in this species.

Simultaneous to the change in shoal area observed during vocalisation playback, captive bigeyes were found to increase their vocalisation rate. Many animals are known to respond both physically and vocally to contact calls of conspecifics^2^. In captive experiments, physically isolated vampire bats presented with conspecific vocal prompts will produce contact calls until reunited with group members^38^. Juvenile black caiman, emit simple contact calls that are used to attract siblings, and playback experiments have led to increased group cohesion and increased vocalisation rates by individuals gathering on or near water banks^4^. In this current study, bigeyes produced over five times the number of calls during vocalisation playback than they did during silent controls, which indicates these vocalisations have communicative value, and the simultaneous change in shoal area suggests they are also likely be related to shoal cohesion.

Contact calls are used over large distances to coordinate group movement in mammals, such as African elephants^7^,^39^ and chimpanzees^3^. In the marine environment, Hawaiian spinner dolphins use different call types to attract individuals depending on their spatial grouping^8^. The simultaneous change in vocal behaviour and group cohesion observed under ambient sound and vocalisation playback are further supported by evidence from recordings of captive fish which show that bigeyes vocalise more at dusk and at night than during the day^18^, which is concurrent with the timing of increased activity of wild bigeyes^23^. Furthermore, the hearing specialisation of this species and the large spatial scale of the active space of the bigeye call, which has a maximum calling distance of 31.6 m, make it an effective cue for a loose shoaling, nocturnal species^18^.

Sound production in fish evolved for the purpose of acoustic signalling^10^ and the convergent evolution of sound production across a variety of fish species highlights the importance of sound for communication. It is thought that all fishes can detect sound and that the auditory mechanisms seen in mammals and birds are modifications of more basic functions found in early fish^40^. The prevalence of contact calls in social mammals, birds, amphibians, and reptiles, and the evidence now found for this nocturnal fish species, highlights the importance of sound as a group cohesion cue, and extends the phylogenetic distribution of contact calls to include teleost fishes. The emerging opportunity for soundscapes to provide a tool for the study of fish communities depends on an adequate inventory of species-specific sounds and the behavioural context in which they are produced^41^. Contact calls add an additional layer to the interpretation of nocturnal reef soundscapes.

## Materials and Methods

Bigeyes were collected by SCUBA divers using soft-meshed nets from a cave on the Leigh coastline (36°17′23.70“S, 174°49′11.97”E), north-east New Zealand, where a large number of bigeyes are known to take refuge during the day. These fish were transported to the nearby Leigh Marine Laboratory where they were held in a large, black polyethylene holding tank (circular 2800 L, 1.5 m in diameter) with flow-through ambient seawater. They were allowed 3 weeks to acclimate to their artificial “cave”, where they were monitored daily and fed 3 times a week. All experiments were carried out in accordance with the University of Auckland’s ethics committee approval number 001150.

### Experimental setup

Individual experiments took place by transferring selected fish into a white polyethylene tank (circular 1500 L, 1.84 m in diameter) supplied with flow-through ambient seawater. A GoPro Hero3 (GoPro Inc.) camera was mounted 1 m above the centre of the tank, below which a Soundtrap 202 hydrophone (frequency response 20 Hz–60 kHz, Oceaninstruments Ltd) was submerged midway in the water column. Digitally recorded sounds were played from an MP3 player connected to a 222 W amplifier and delivered into the tank via a J9 speaker (Naval Undersea Warfare Center, Underwater Sound Reference Division, Newport, US) (projector 40 Hz to 20 kHz) suspended midway in the water column at the margin of the tank and projecting into the tank. All external light was eliminated from the experimental tank with illumination provided by a small number of dim red LED lights.

A recording was taken in the experimental tank while fish were absent and water flow was off and the minimum power level of ambient noise was measured at an average received level of 100 dB dB re 1 μPa, due to transferred noise from the nearby coast, water pumps and electrical equipment.

### Sound files

**Figure 6:**
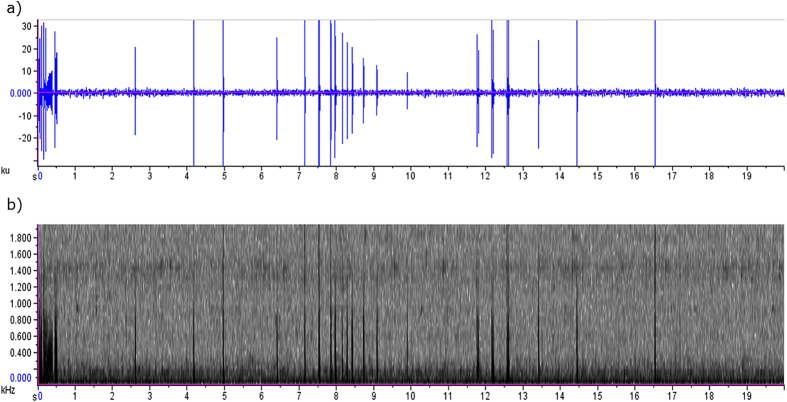
Waveform (a) and spectrogram (b) of 20 second sample from recording of bigeye vocalisations from captive fish and used in the vocalisation playback experiment.

Two pre-recorded sound files were used in this experiment: ambient reef sound and bigeye vocalisations. The ambient reef sound file consisted of a 10 minute sound clip taken from a recording of North Reef in the Cape Rodney to Okakari Point Marine Reserve nearby to where the fish for the experiment were collected. This recording did not contain any bigeye vocalisations and is representative of ambient sound experienced by these fish in their natural habitat. The 10 minute bigeye vocalisation file was composed of repeated 20 second segments (Fig. 6) of bigeye calls recorded during experiments on captive fish reported in^18^.

Sound files were played at averaged received levels well above the tank’s ambient sound level. Average received levels were calculated from recordings taken at 12 points over a grid laid out within the tank, with the sound level adjusted via the output control on the amplifier. Ambient reef sound was played at average received levels in the tank of 125 dB, 130 dB and 135 dB re 1 μPa, while the bigeye vocalisation track was played at an average received level of 135 dB re 1 uPa.

### Playback experiments

Four groups of 24 randomly selected fish were carefully transferred from the holding tank to the experimental tank and left for at least 24 hours. Experiments began at dusk the following day, at which time the water flow to the tank was turned off to reduce background noise. Each group of fish were exposed to only one received sound level per night, and were only exposed to the three received ambient levels and vocalisation playback once, with at least 24 hours separating the start of each experiment to eliminate the effects of previous sound exposure.

### Ambient Sound playback

Ambient reef sound was played at one of the three received levels for 10 minutes followed by 10 minutes of silence (sound on and sound off), which repeated seven times. The hydrophone constantly recorded all ambient, playback and biological sound while the GoPro took an overhead digital image of the tank every 60 seconds enabling the position of all individual fish to be identified.

### Vocalisation playback

Following the same methods as the ambient sound playback, the four groups of fish were exposed to vocalisation playback at least 24 hours after the final ambient sound experiment.

### Data analyses

Shoal area was determined as the percentage of the total two-dimensional area of the tank occupied by the shoal of fish and was taken as a measure of group cohesion. Digital images were analysed using ImageJ (http://imagej.nih.gov/ij/) in which the outer edges of the outer most members of the shoal were connected and the area enclosed within this margin was calculated as a percentage of total available tank area. This method was used by Domenici, *et al.*^42^ who found it to be as effective a measure of group cohesion as the total three-dimensional area, or volume, of the shoal. Average shoal area was calculated for 70 images taken during silent control periods and while playback sound was on.

Sound recordings from the tank during the experimental period were edited in Audacity using a low pass filter and then run through PAMGuard (www.pamguard.org), which was configured to count the number of bigeye calls. The number of calls was determined for each of the seven sound on and sound off periods and then a mean calculated for both the sound on and sound off periods. Bigeye calls recorded included a range of fused and unfused pop sounds. Radford, *et al.*^18^ defined unfused pops as those separated by at least 100 ms, but for maximum accuracy in PAMGuard this was set to 150 samples with a maximum click length of 1500 samples. For this reason, some fused calls were counted as single pops, but this approach was consistent across all sound recordings that were analysed.

For vocalisation playback experiments, the number of calls emitted from the speaker in each 10 minute segment (1717) was subtracted from the number of calls detected in the experimental recordings to determine the number of calls produced by the fish in the tank.

### Statistical analysis

Paired t-tests were used to determine any differences between sound on and sound off periods for the shoal area and for the number of bigeye calls during both ambient sound and bigeye vocalisation playback experiments. Where non-normality of the data was detected a priori Wilcoxon signed rank test was performed. Data are presented as means ± S.E.

## Additional Information

**How to cite this article**: van Oosterom, L. *et al.* Evidence for contact calls in fish: conspecific vocalisations and ambient soundscape influence group cohesion in a nocturnal species. *Sci. Rep.* **6**, 19098; doi: 10.1038/srep19098 (2016).
